# Supplementary material for: Blood methylation pattern reflects epigenetic remodelling in adipose tissue after bariatric surgery
Source: eBioMedicine. 2024 Jul 13;106:105242. doi: 10.1016/j.ebiom.2024.105242 (PMC11284569; doi:10.1016/j.ebiom.2024.105242)
Supplement: Supplementary Figures [file mmc3.pdf]

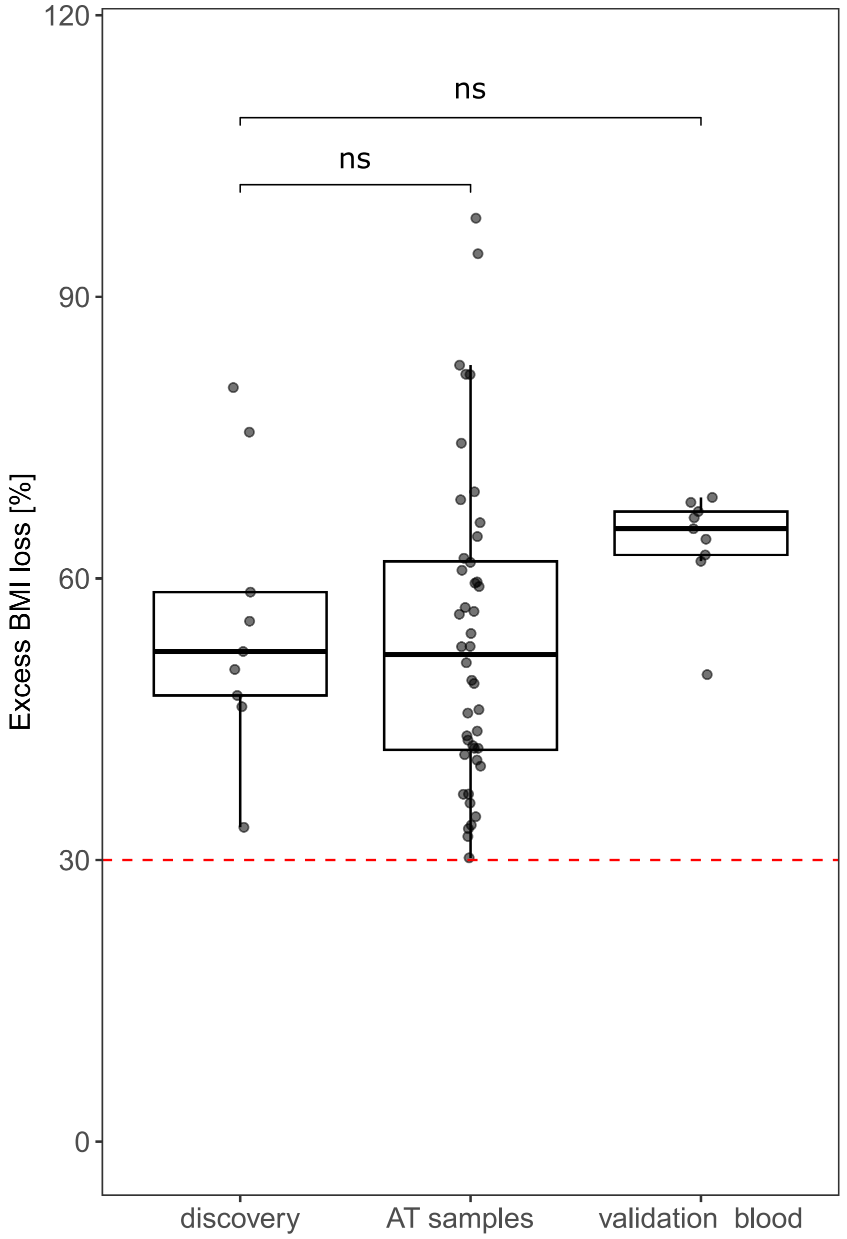

Supplemental Figure S1| Excess BMI loss (%) over all cohorts. Boxplots show Excess BMI Loss (EBL, %) in discovery cohort (N = 9), adipose tissue mRNA expression cohort (AT samples, N = 44), and blood validation cohort (validation blood, N = 9). The dashed red line indicates the inclusion cut-off criteria of at least 30% EBL. Significance levels of comparisons were calculated by Wilcoxon rank sum test for unpaired samples. ns: not significant ($P \geq 0.05$).

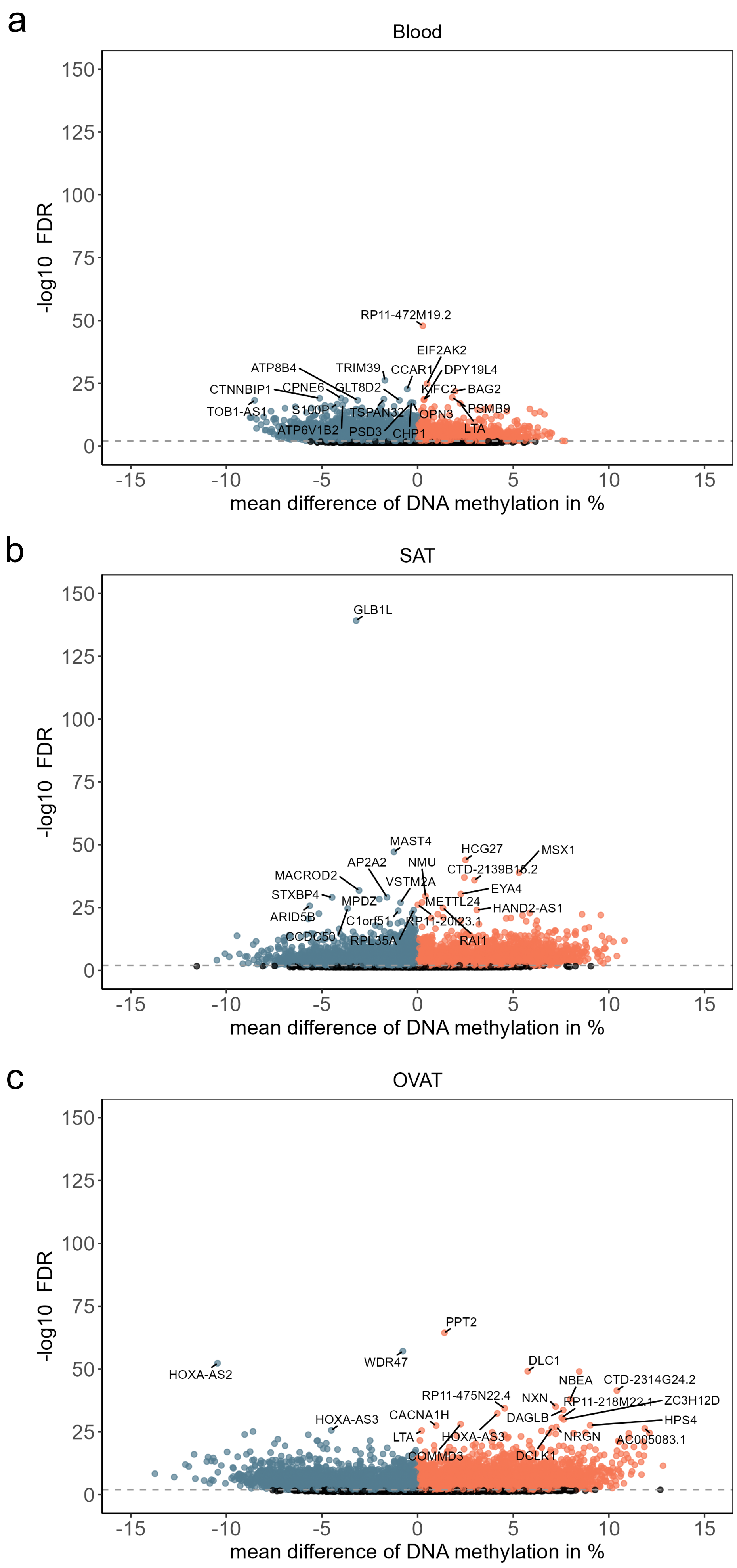

Supplemental Figure S2| Differentially methylated regions (DMRs) of all tissues. Volcano plots of DMRs in a) blood, b) subcutaneous adipose tissue (SAT), and c) omental visceral adipose tissue (OVAT). Blue points represent hypomethylated DMRs (mean methylation difference < 0, FDR < 0.01) and red points represent hypermethylated DMRs (mean methylation difference > 0, FDR < 0.01) after surgery. Black points show DMRs with FDR between 0.01 and 0.05. Top 20 DMRs (ranked by FDR) are labelled with the annotated gene.

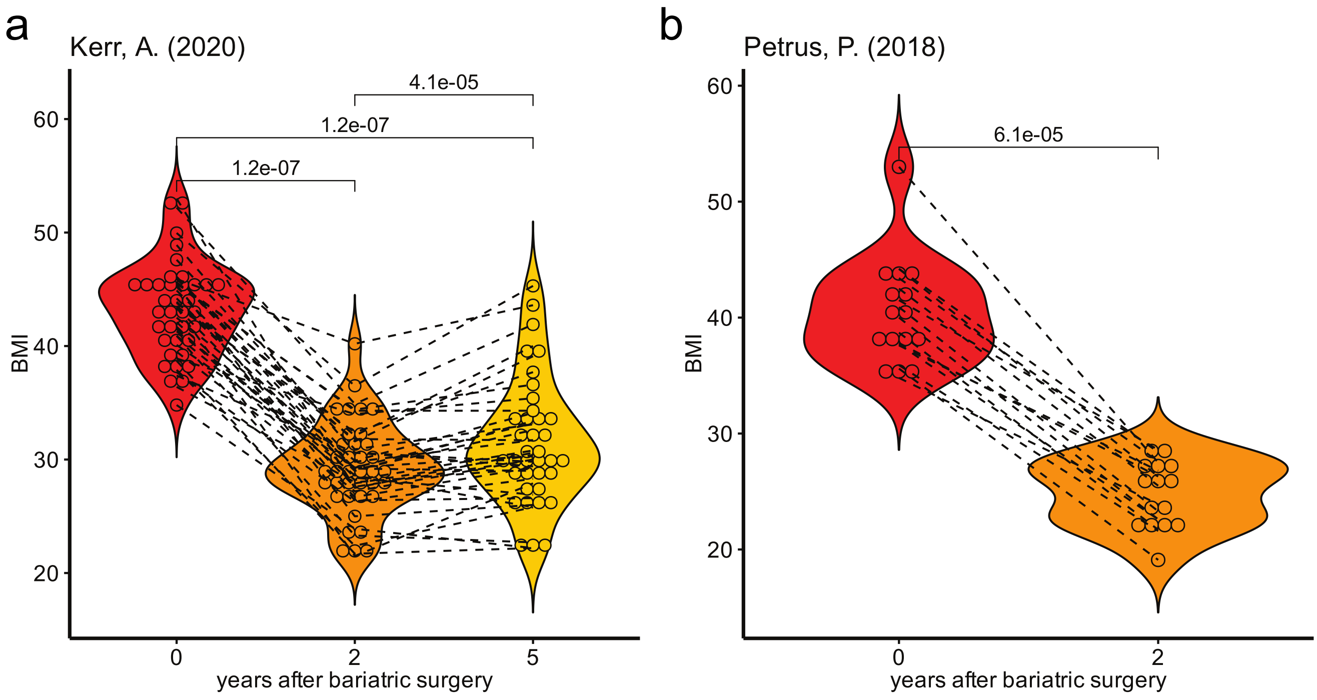

Supplemental Figure S3| Weight loss in other gene expression studies after bariatric surgery. Violin plots indicate weight loss after bariatric surgery in both studies of a) Kerr et al. (2020, PMID: 32406570) and b) Petrus et al. (2018, PMID: 30332637). Depicted *P*-Values are calculated by Wilcoxon signed rank test for paired data.

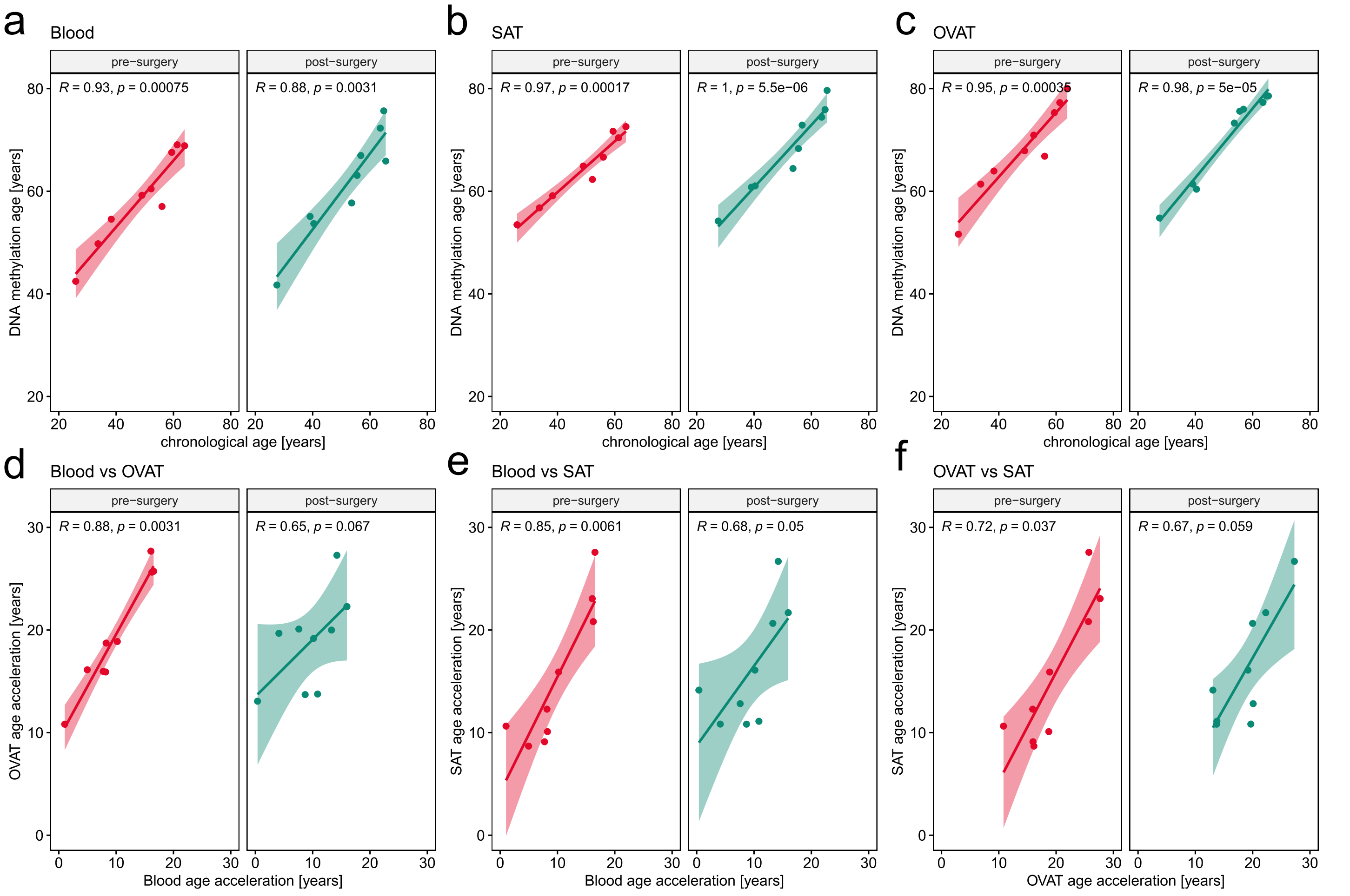

Supplemental Figure S4| Correlation of DNAmAge with chronological age and ageAcc between tissues. a-c) Scatter plots show correlation of DNA methylation age and chronological age (ageAcc) for pre and post-surgery in a) blood, b) subcutaneous adipose tissue (SAT), and c) omental visceral adipose tissue (OVAT). d-f) Scatter plots show correlation of age acceleration (ageAcc) between tissues for pre and post-surgery. Correlations were calculated for ageAcc between d) blood and OVAT, e) blood and SAT, and f) SAT and OVAT. For all correlations Spearman correlation coefficient (R = rho) and nominal significance (p) of correlation are depicted and a linear regression line is added.

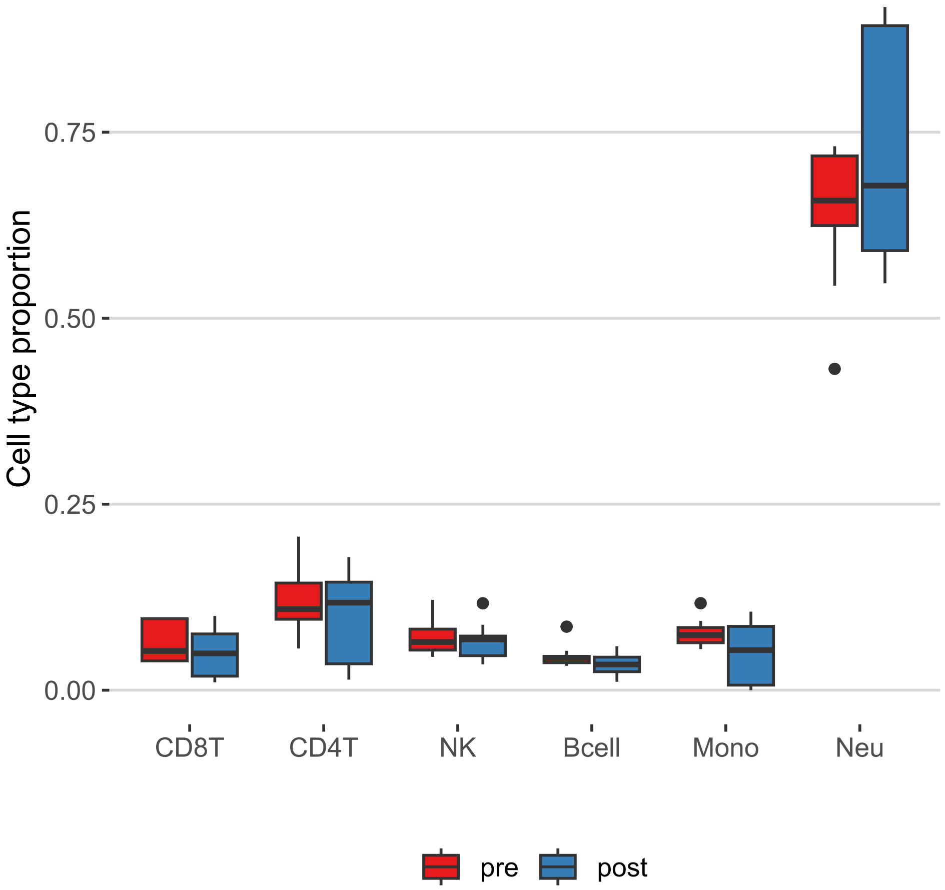

Supplemental Figure S5| Cell type distribution. Boxplots show proportions of cell type populations (CD8 T-lymphocytes (CD8T), CD4 T-lymphocytes (CD4T), natural killer cells (NK), B-cells (Bcell), monocytes (Mono), and neutrophils (Neu)) between pre-surgical (red) and post-surgical (blue) methylation pattern based on Illumina 850K methylation data.

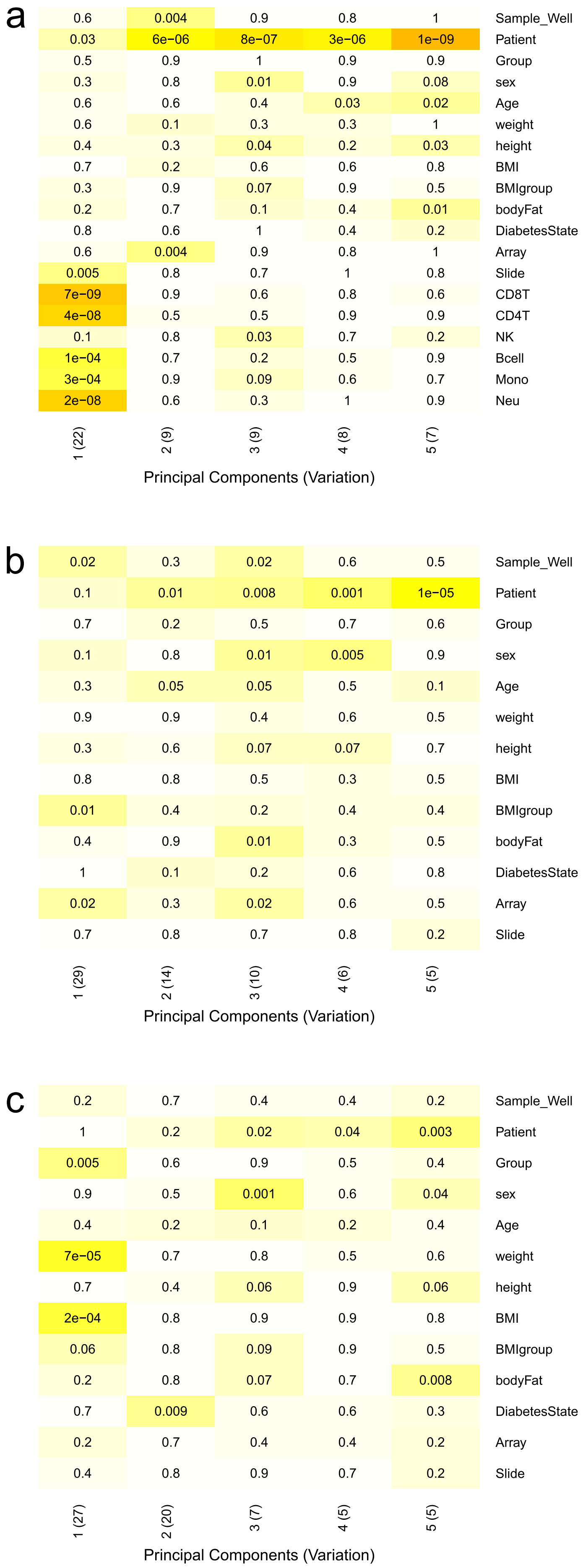

Supplemental Figure S6| Principal components of unadjusted DNA methylation data. Heatmaps show significance level (*P*-value) of associations of top five principal components of normalised EPIC array data with sample annotations in a) blood, b) subcutaneous adipose tissue, and c) omental visceral adipose tissue samples before adjustment.

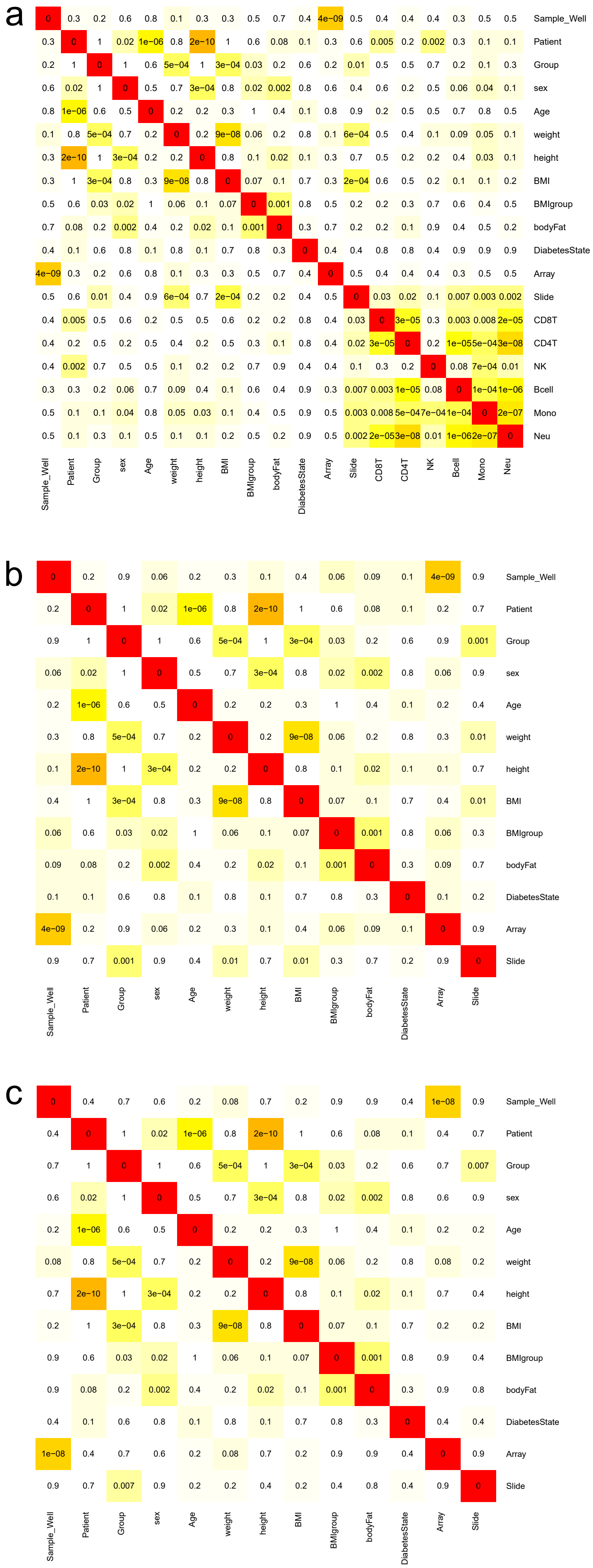

Supplemental Figure S7| Correlation of sample annotations. Correlation matrices show interrelation of sample annotations in a) blood, b) subcutaneous adipose tissue, and c) omental visceral adipose tissue samples in order to identify confounding factors. Significance level (*P*-values) of correlations are depicted.

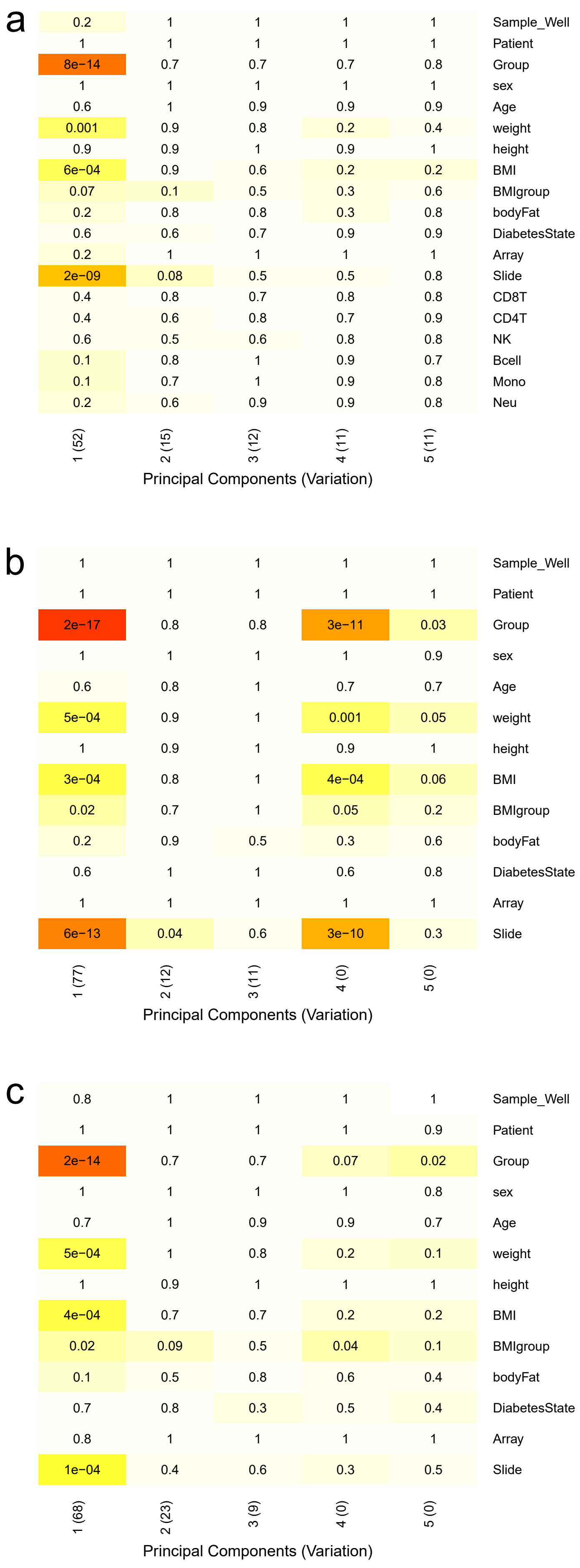

Supplemental Figure S8| Principal components after adjustment of DNA methylation data. Heatmaps show significance level (*P*-value) of associations of top five principal components of normalised EPIC array data adjusted for array and patient factor in a) blood, b) subcutaneous adipose tissue, and c) omental visceral adipose tissue samples.

a

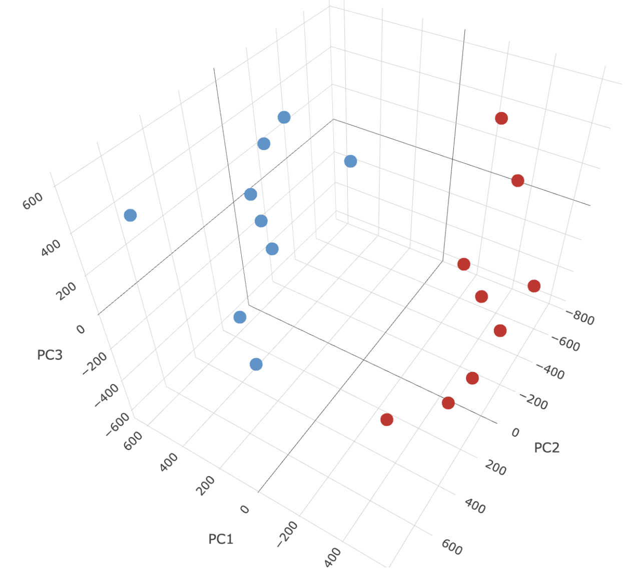

b

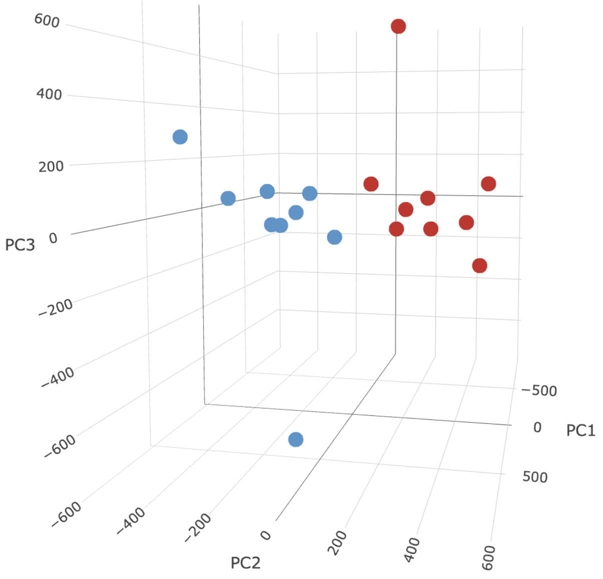

c

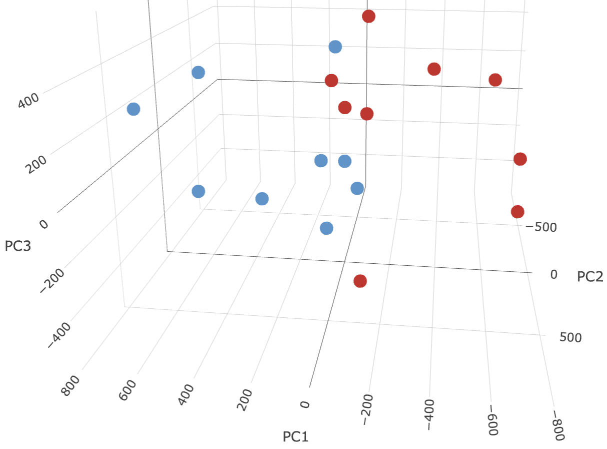

Supplemental Figure S9| Visualisation of Principal Component Analysis (PCA) after adjustment. Three-dimensional PCA plots of normalized EPIC array data adjusted for array and patient factor in a) blood, b) subcutaneous adipose tissue, and c) omental visceral adipose tissue samples show separation of pre (red)- vs post-surgery (blue) samples. PC: principal component.
